# Balance of interstitial flow magnitude and vascular endothelial growth factor concentration modulates three-dimensional microvascular network formation

**DOI:** 10.1063/1.5094735

**Published:** 2019-07-30

**Authors:** Yoshinori Abe, Masafumi Watanabe, Seok Chung, Roger D. Kamm, Kazuo Tanishita, Ryo Sudo

**Affiliations:** 1 School of Integrated Design Engineering, Graduate School of Science and Technology, Keio University, Yokohama 223-8522, Japan; 2 School of Mechanical Engineering, Korea University, Seoul 02841, South Korea; 3 Departments of Mechanical Engineering and Biological Engineering, Massachusetts Institute of Technology, Boston, Massachusetts 02139, USA; 4 Department of System Design Engineering, Keio University, Yokohama 223-8522, Japan

## Abstract

Hemodynamic and biochemical factors play important roles in critical steps of angiogenesis. In particular, interstitial flow has attracted attention as an important hemodynamic factor controlling the angiogenic process. Here, we applied a wide range of interstitial flow magnitudes to an *in vitro* three-dimensional (3D) angiogenesis model in a microfluidic device. This study aimed to investigate the effect of interstitial flow magnitude in combination with the vascular endothelial growth factor (VEGF) concentration on 3D microvascular network formation. Human umbilical vein endothelial cells (HUVECs) were cultured in a series of interstitial flow generated by 2, 8, and 25 mmH_2_O. Our findings indicated that interstitial flow significantly enhanced vascular sprout formation, network extension, and the development of branching networks in a magnitude-dependent manner. Furthermore, we demonstrated that the proangiogenic effect of interstitial flow application could not be substituted by the increased VEGF concentration. In addition, we found that HUVECs near vascular sprouts significantly elongated in >8 mmH_2_O conditions, while activation of Src was detected even in 2 mmH_2_O conditions. Our results suggest that the balance between the interstitial flow magnitude and the VEGF concentration plays an important role in the regulation of 3D microvascular network formation *in vitro*.

## INTRODUCTION

Angiogenesis is the phenomenon that new blood vessels are formed from existing blood vessels, which occurs under physiological and pathological conditions such as tissue regeneration, ischemic disorder, and tumor growth.^1–3^ Elucidation of the angiogenic process is one of the major challenges to construction of functional microvascular networks, vascularization of tissue-engineered constructs, and the control of pathological angiogenesis. The process of angiogenesis includes endothelial cell (EC) migration, proliferation, and degradation of the extracellular matrix (ECM) in response to angiogenic stimuli.^4^ Particularly, biochemical and hemodynamic factors have been shown to play important roles in critical steps of the angiogenic process.^5,6^

Various biochemical factors which promote microvascular network formation have been recognized.^1,7,8^ In particular, the vascular endothelial growth factor (VEGF) is a major angiogenic factor, which plays important roles in vascular sprouting and microvascular network formation.^9–11^ The VEGF concentration gradient induces generation of endothelial tip cells which directionally branch from existing vessels, leading rear ECs (stalk cells) to the direction of the VEGF concentration gradient. The protrusion of subcellular pseudopodial branches on endothelial tip cells can sense VEGF concentration gradients.^11^ These findings suggest that the precise control of VEGF supply is critical to the control of three-dimensional (3D) network formation.

In addition to biochemical factors, hemodynamic factors become increasingly important in regulation of angiogenic processes. ECs covering the luminal surface of blood vessels are constantly exposed to hemodynamic factors such as fluid shear stress and pressure due to blood shear flow and plasma interstitial flow.^5,12^ It has long been known that shear stress plays important roles in critical steps of angiogenic processes such as orientation, cytoskeletal reorganization, migration, and angiogenic gene expression in ECs.^13–16^ Furthermore, microvascular network formation was enhanced depending on shear stress magnitude in both *in vivo* and *in vitro* experiments.^17,18^ Not only shear flow but also interstitial flow has been shown to affect microvessel formation. Helm *et al.*^19^ reported that interstitial flow, which was applied to ECs embedded in 3D gels, synergistically promoted capillary morphogenesis with VEGF. In addition, Hernández Vera *et al.*^20^ applied interstitial flow from the apical to the basal side of an endothelial monolayer, which enhanced capillary morphogenesis. These results suggested that interstitial flow is an important regulator of capillary morphogenesis.

Microfluidic devices have been used for development of *in vitro* 3D angiogenesis models that mimic *in vivo* microenvironments. In particular, the precise control of fluid in a microfluidic device led to the investigation of angiogenic processes affected by directional interstitial flow with monitoring angiogenic cell behaviors in real time. Vickerman and Kamm^21^ reported that directional interstitial flow, which was applied from the basal side to the apical side of an endothelial monolayer, promoted capillary morphogenesis while the adverse flow direction had no effects. Song and Munn^22^ reported that interstitial flow enhanced sprouting morphogenesis with the formation of more filopodia extending against the flow direction. In addition, Shirure *et al.*^23^ reported that vascular formation was promoted in the upstream of interstitial flow. However, they used an angiogenesis model where endothelial cells were embedded in gels. In this condition, interstitial flow was passing over the abluminal surface of blood vessels, while transmural interstitial flow, which is a flow across an endothelial monolayer, is also important in an angiogenesis model. Galie *et al.*^24^ reported that transmural flow guided preferential sprouting toward paths of draining interstitial flow. These results suggest that microvascular formation can be controlled by transmural interstitial flow. However, it remains unclear whether transmural interstitial flow magnitude can be optimized for controlling 3D network formation in combination with various VEGF concentrations.

Here, we investigated the effect of interstitial flow magnitude on 3D microvascular network formation. Specifically, we utilized microfluidic devices to control a series of interstitial flow magnitudes, which was generated by 2, 8, and 25 mmH_2_O pressure differences. Although there is increasing evidence that the interaction between ECs and stromal cells, such as pericytes and fibroblasts, is important during vascular formation,^25–29^ we used a simple *in vitro* model which is composed of only ECs to focus on the direct effect of interstitial flow on the angiogenic response of ECs. First, we analyzed the process of microvascular network formation in the series of interstitial flow conditions. We then investigated microvascular network formation in the combination of interstitial flow and the VEGF concentration focusing on VEGF flux. Furthermore, we explored a mechanism that can explain the effect of interstitial flow on angiogenesis.

## RESULTS

### *In vitro* microvascular network formation in a series of interstitial flow magnitude

**FIG. 1.**
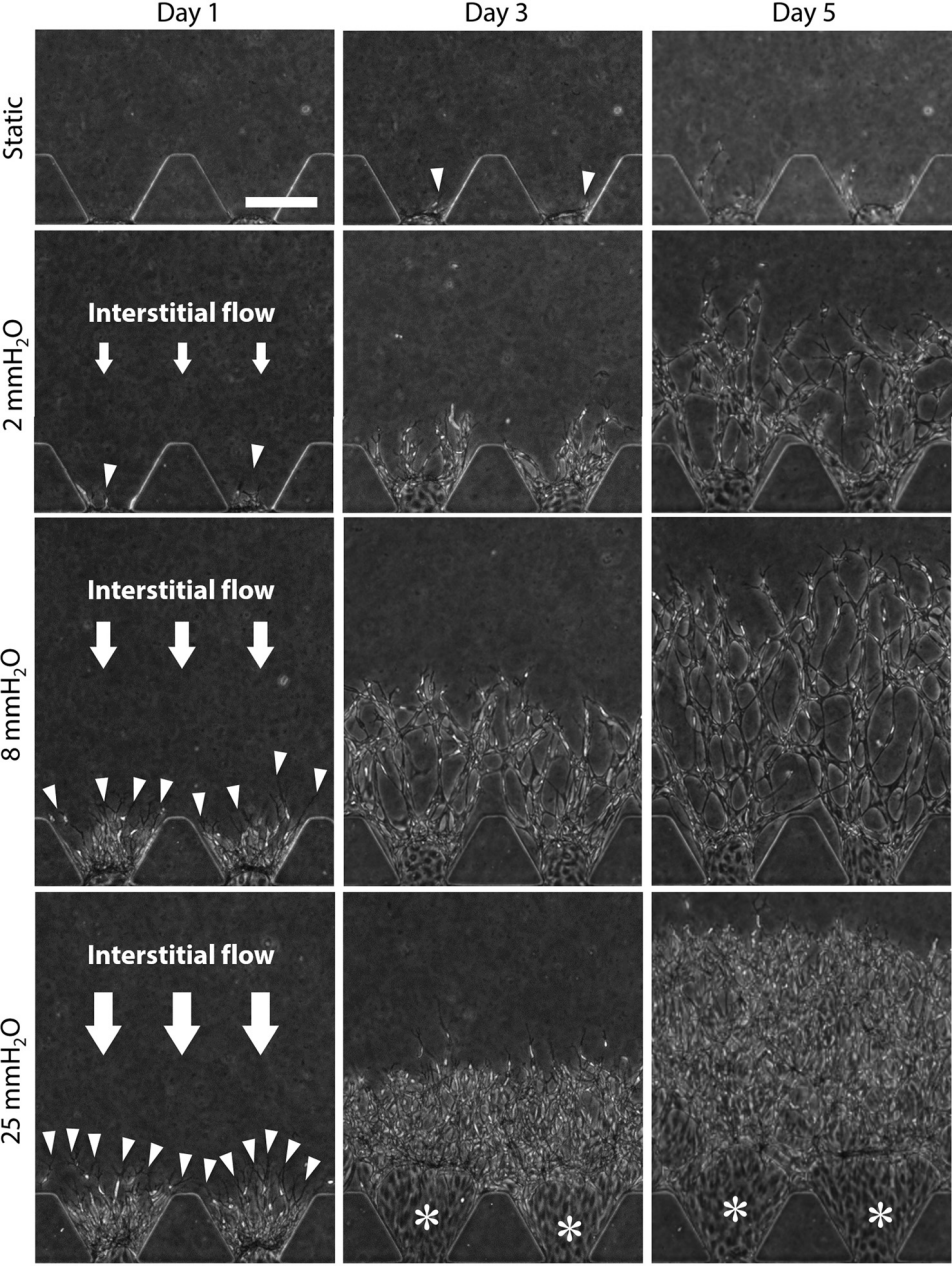
The process of endothelial network formation in a series of interstitial flow. The representative phase-contrast images of HUVECs cultured in 2, 8, and 25 mmH_2_O conditions and static conditions. The arrowheads indicate vascular sprouts. The asterisks indicate regions of dissolved collagen gel. Scale bar, 200 *μ*m. All images were taken at the same magnification.

HUVECs were cultured in a series of interstitial flow magnitude, 2, 8, and 25 mmH_2_O conditions corresponding to the mean velocities of 0.07, 0.7, and 4.3 μm/s during the first 24 h of interstitial flow experiments, respectively (Table I, for details, see the supplementary material), while cells were also cultured in static conditions as control experiments. Cells were monitored by phase-contrast microscopy until day 5 to clarify the effect of interstitial flow magnitude on 3D network formation. The representative phase-contrast images showed that HUVECs formed vascular sprouts extending into collagen gel on day 1 only in interstitial flow conditions (arrowheads, Fig. 1). In contrast, vascular sprouts were not observed in static conditions on day 1, although a few sprouts were formed on day 3 (arrowheads, Fig. 1), which then regressed by day 5. In interstitial flow conditions, vascular sprouts extended against the flow direction, which were then developed into microvascular networks during days 3–5. Microvascular network formation was promoted with increasing magnitude of interstitial flow. However, collagen gel was excessively degraded in regions near the endothelial monolayer especially in 25 mmH_2_O conditions (asterisks, Fig. 1).

### Effect of interstitial flow magnitude on early stages of microvascular formation

**FIG. 2.**
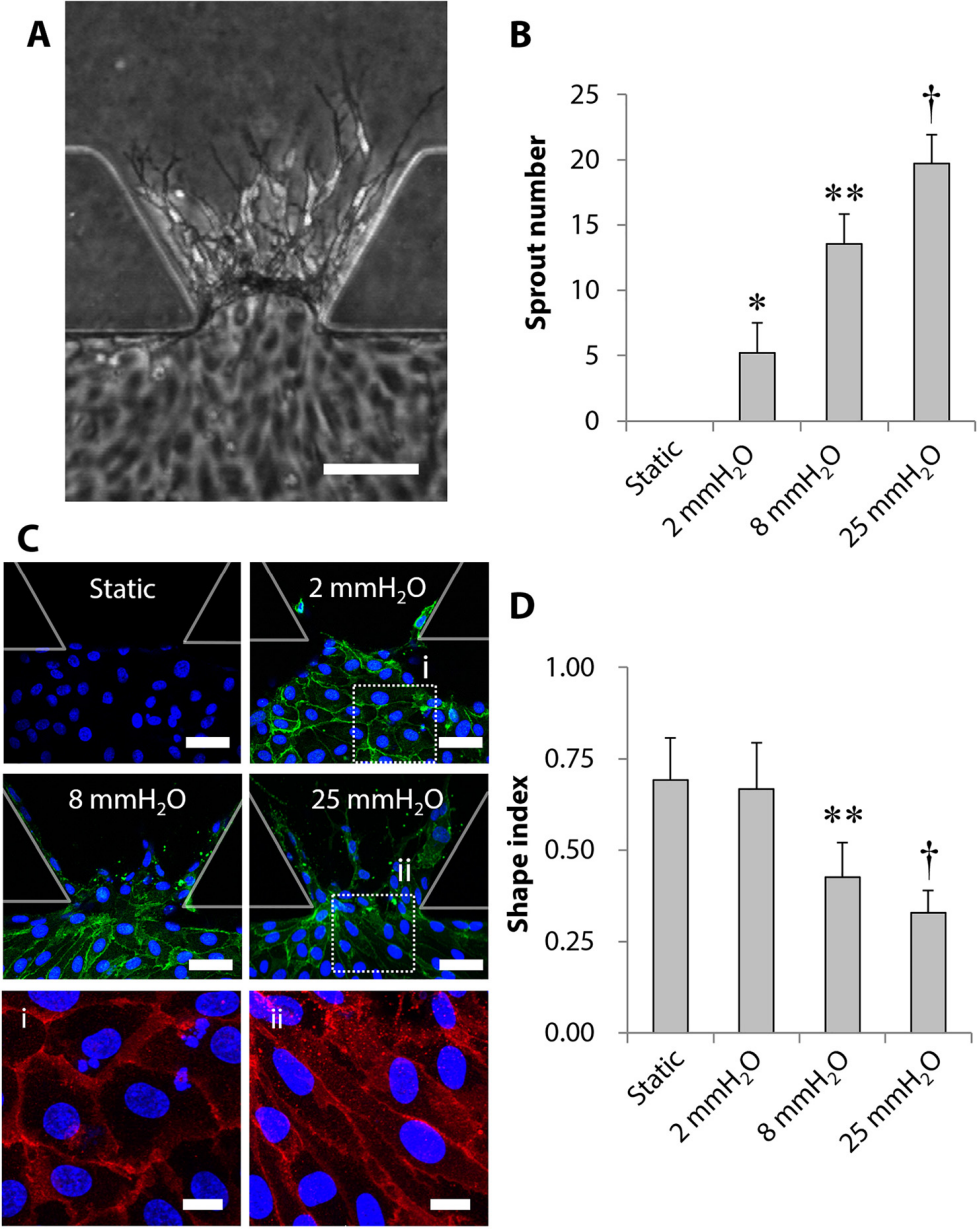
Effect of interstitial flow magnitude on early stages of vascular formation. (a) The representative phase-contrast images of vascular sprouts on day 1 in 8 mmH_2_O conditions. (b) Quantitative analysis of the number of vascular sprouts. Data = mean± SD; n = 24. *P <* *0.05 vs static. **P <* *0.05 vs 2 mmH_2_O. ^†^*P *<* *0.05 vs the other conditions. (c) Immunofluorescence images of Src (green) and nuclei (DAPI, blue) cultured in the series of interstitial flow conditions. Rectangles (i) and (ii) were enlarged to show the alignment of HUVECs in an endothelial monolayer, where cell–cell borders were visualized with PECAM. (d) The shape index was calculated to quantify the alignment of HUVECs. Data = mean ± SD; n = 24. **P <* *0.05 vs 2 mmH_2_O. ^†^*P *<* *0.05 vs the other conditions. Scale bars, 100 *μ*m (a), 50 *μ*m (c), and 15 *μ*m (i) and (ii).

Next, we analyzed the effect of interstitial flow on HUVECs focusing on early stages of microvascular network formation because the effect of interstitial flow was observed even on day 1 [Fig. 2(a)]. First, the number of vascular sprouts was quantified, which showed that the sprout number increased with increasing magnitude of interstitial flow [Fig. 2(b)]. Sprout numbers in 8 and 25 mmH_2_O conditions were 2.6 and 3.8 times larger than that in 2 mmH_2_O conditions, respectively.

Second, we investigated the expression of pSrc, which might be involved downstream of integrin activation by interstitial flow.^32^ Immunofluorescence images clearly showed that Src expression was observed in interstitial flow conditions, whereas no expression was detected in static conditions [Fig. 2(c)]. The Src expression was detected even in the lowest interstitial flow magnitude, 2 mmH_2_O conditions, and no significant difference of the expression was observed among 2, 8, and 25 mmH_2_O conditions.

Third, we focused on the alignment of HUVECs in a monolayer formed near the collagen gel region. Immunofluorescence images revealed that the HUVECs elongated toward the gel region in 25 mmH_2_O conditions, while no elongation was observed in 2 mmH_2_O conditions [i–ii, Fig. 2(c)]. This morphological change was quantified by calculating the shape index [Fig. 2(d)]. These results revealed that HUVECs in a monolayer significantly elongated in 8 and 25 mmH_2_O conditions, while those in 2 mmH_2_O conditions were similar to those in static conditions.

### Effect of interstitial flow magnitude on 3D network formation

Since we confirmed that interstitial flow magnitude had significant effects on early stages of microvascular formation such as vascular sprout formation, we then focused on the following stages, network extension and the process of branching network formation.

**FIG. 3.**
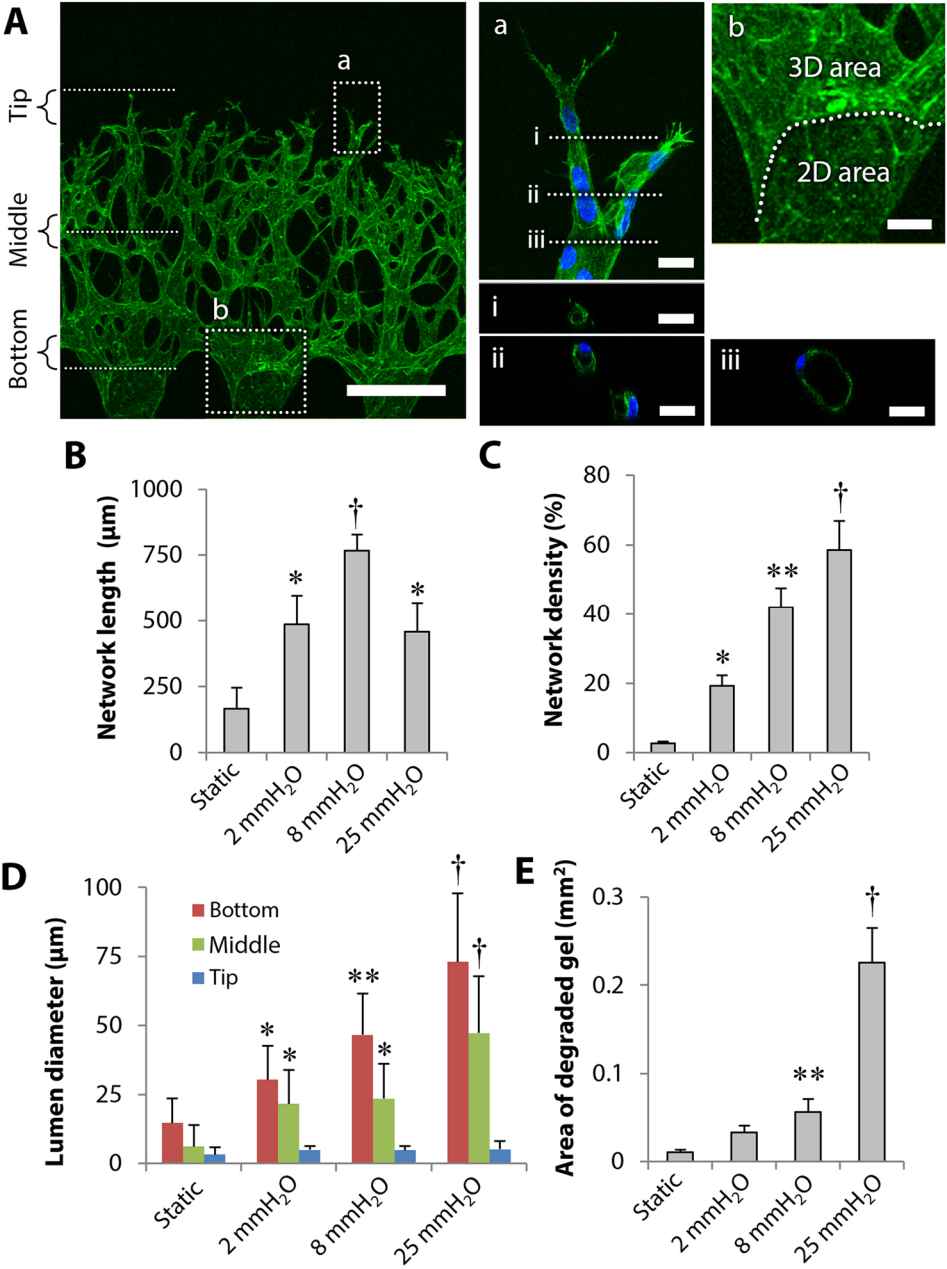
Effect of interstitial flow magnitude on 3D network formation. (a) The representative fluorescence images of 3D networks formed on day 5 in 8 mmH_2_O conditions (actin in green and nuclei in blue). Tip, middle, and bottom regions of networks were focused for lumen diameter measurements. Rectangles *a* and *b* were enlarged to show tip (a) and bottom (b) regions of networks. Cross-sectional images (i)–(iii) of the network showed continuous lumens. Scale bars, 300 *μ*m (a), 20 *μ*m (a, i, ii, iii), and 50 *μ*m (b). (b)–(d) Quantitative analysis of the network length (b), density (c), lumen diameter (d), and area of degraded gel (e). Data = mean ± SD; n = 8–10 (b), (c), and (e), n = 24 (d). *P <* *0.05 vs static. **P <* *0.05 vs 2 mmH_2_O. †*P *<* *0.05 vs the other conditions except for 25 mmH_2_O.

First, the structure of 3D microvascular networks was observed using a confocal microscope. The representative projected images showed that HUVECs formed 3D network structures on day 5 in 8 mmH_2_O conditions [Fig. 3(a)]. Cross-sectional images revealed that HUVECs formed 3D microvasculatures with continuous lumens from an endothelial monolayer to the network tip [i–iii, Fig. 3(a)]. Although HUVECs formed 3D microvasculatures in interstitial flow conditions, collagen gel was excessively degraded in regions near the endothelial monolayer, resulting in the formation of a “3D area” and a “2D area” at the entrance to the microvascular networks [b, Fig. 3(a)].

We further analyzed microvascular networks formed on day 5, focusing on the length, density, lumen diameter, and area of degraded gel. Quantitative analysis revealed that the length of microvascular networks in the 3D area increased by the application of interstitial flow, and the maximum network length was observed in 8 mmH_2_O conditions [Fig. 3(b)]. The network length in 25 mmH_2_O conditions decreased due to degradation of collagen gel, compared to the length in 8 mmH_2_O conditions [Fig. 3(b)]. The network density significantly increased with increasing magnitude of interstitial flow [Fig. 3(c)]. The lumen diameter of the 3D networks was measured within 100-*μ*m regions at the tip, middle, and bottom of networks, respectively [e.g., Tip, Middle, Bottom, Fig. 3(a)]. The results revealed that the lumen diameter significantly increased in response to interstitial flow magnitude at the bottom and middle of networks, while no significant difference was detected at network tips [Fig. 3(d)].

Finally, we focused on the formation of the 2D area shown in Fig. 3(a). We assumed that the 2D area appeared due to degradation of collagen gel by HUVECs whose MMP activity can be enhanced by interstitial flow application. The area of degraded gel significantly increased with increasing magnitude of interstitial flow. In particular, the area of degraded gel in 25 mmH_2_O conditions was 4.0 times larger than that in 8 mmH_2_O conditions [Fig. 3(e)].

### Effect of the interstitial flow magnitude and VEGF concentration on 3D network formation

**FIG. 4.**
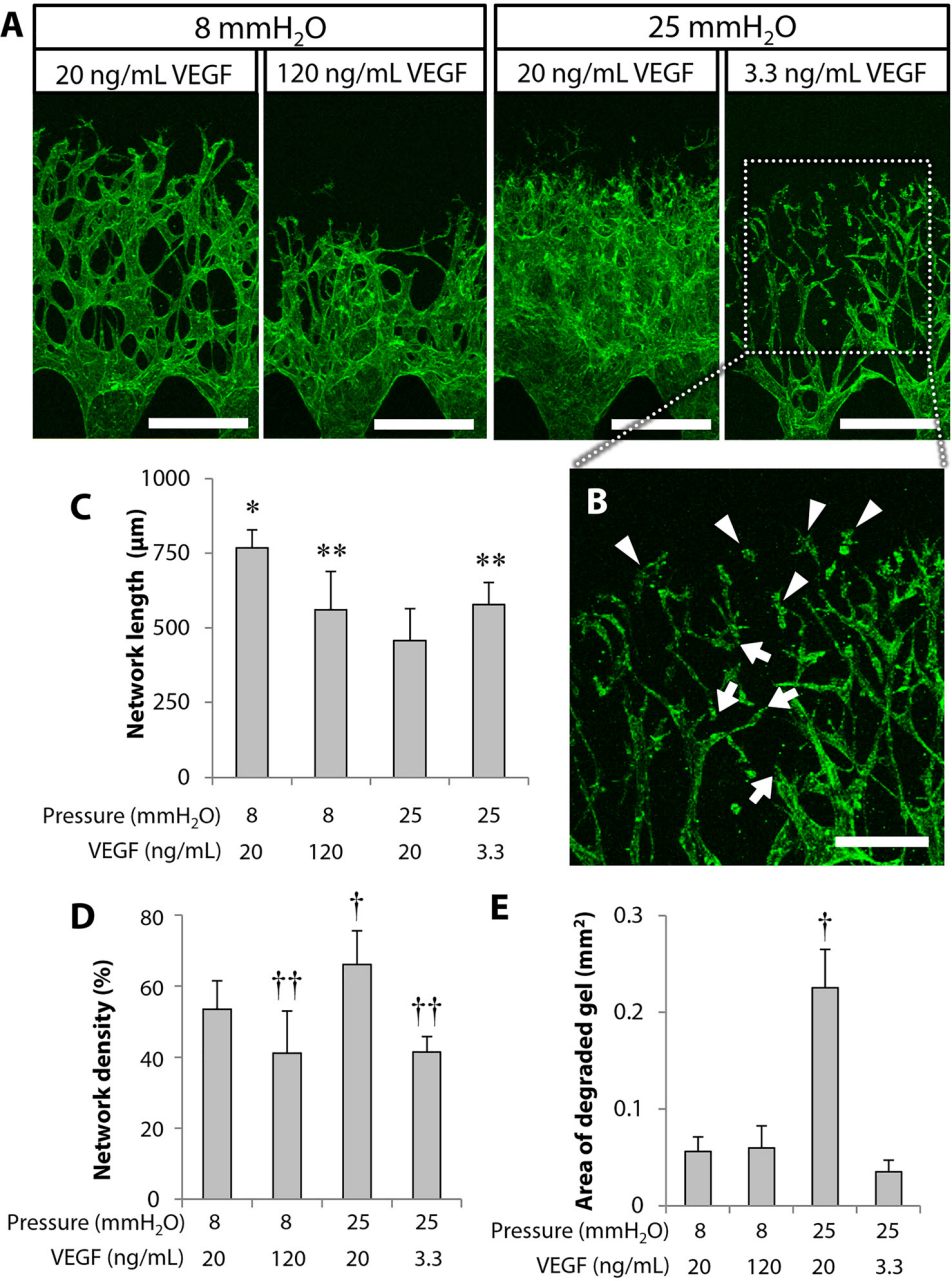
Effect of VEGF flux on 3D network formation. (a) The representative 3D projected images of endothelial networks on day 5. Actin filaments were stained in green. (b) An enlarged image shown in the rectangle in (a). The arrowheads indicate the cells detached from network tips, while the arrows indicate separation of endothelial networks. (c)–(e) Quantitative analysis of the network length and density and area of degraded gel. Data = mean ± SD; n = 15–33 (c), n = 8–10 (d) and (e). *P <* *0.05 vs the other conditions. **P <* *0.05 vs 25 mmH_2_O with 20 ng/ml VEGF. †*P *<* *0.05 vs the other conditions except for 25 mmH_2_O with 20 ng/ml VEGF. ††*P *<* *0.05 vs the other conditions except for 25 mmH_2_O with 20 ng/ml VEGF. Scale bars, 300 *μ*m (a) and 150 *μ*m (b).

To further investigate the promoting effect of interstitial flow on microvascular network formation, we focused on VEGF flux. We assumed that HUVECs consumed most of the VEGF from the medium supplemented at 20 ng/ml because of the report that the angiogenic effects of VEGF were dose-dependent in the range of 1–100 ng/ml.^33^ Therefore, the angiogenic response of HUVECs can be dependent on VEGF flux. Interstitial flow velocity in 25 mmH_2_O conditions was ∼6 times higher than that in 8 mmH_2_O conditions (Table I), suggesting that the VEGF flux in 25 mmH_2_O conditions with 20 ng/ml VEGF corresponds to the VEGF flux in 8 mmH_2_O conditions with 120 ng/ml VEGF. Therefore, we tested 120 ng/ml VEGF in 8 mmH_2_O conditions. However, microvascular networks formed on day 5 in 8 mmH_2_O conditions with 120 ng/ml VEGF were not equivalent to those in 25 mmH_2_O conditions with 20 ng/ml VEGF [Fig. 4(a)] although these two conditions are equivalent in terms of VEGF flux. Microvascular network formation was attenuated in 8 mmH_2_O conditions with 120 ng/ml VEGF compared to 25 mmH_2_O conditions with 20 ng/ml VEGF. The increased VEGF in 8 mmH_2_O conditions appeared to affect the enlargement of vasculatures. In addition, the VEGF concentration in 25 mmH_2_O conditions was decreased to be 3.3 ng/ml, which is one-sixth of the initial concentration, 20 ng/ml VEGF. Microvascular network formation was attenuated in 25 mmH_2_O conditions with 3.3 ng/ml VEGF, which was not equivalent to that in 8 mmH_2_O conditions with 20 ng/ml VEGF [Fig. 4(a)].

We also found that some cells were detached from network tips in 25 mmH_2_O conditions with 3.3 ng/ml VEGF [arrowheads, Fig. 4(b)], resulting in separation of endothelial networks [arrows, Fig. 4(b)]. This caused decreased network length and density compared to 8 mmH_2_O conditions with 20 ng/ml VEGF [Figs. 4(c) and 4(d)]. However, interstitial flow appeared to enhance the migration of endothelial tip cells. Finally, the area of degraded gel was quantified in a series of VEGF flux conditions. The results showed that the area of degraded gel was significantly higher only in 25 mmH_2_O conditions with 20 ng/ml VEGF [Fig. 4(e)].

**FIG. 5.**
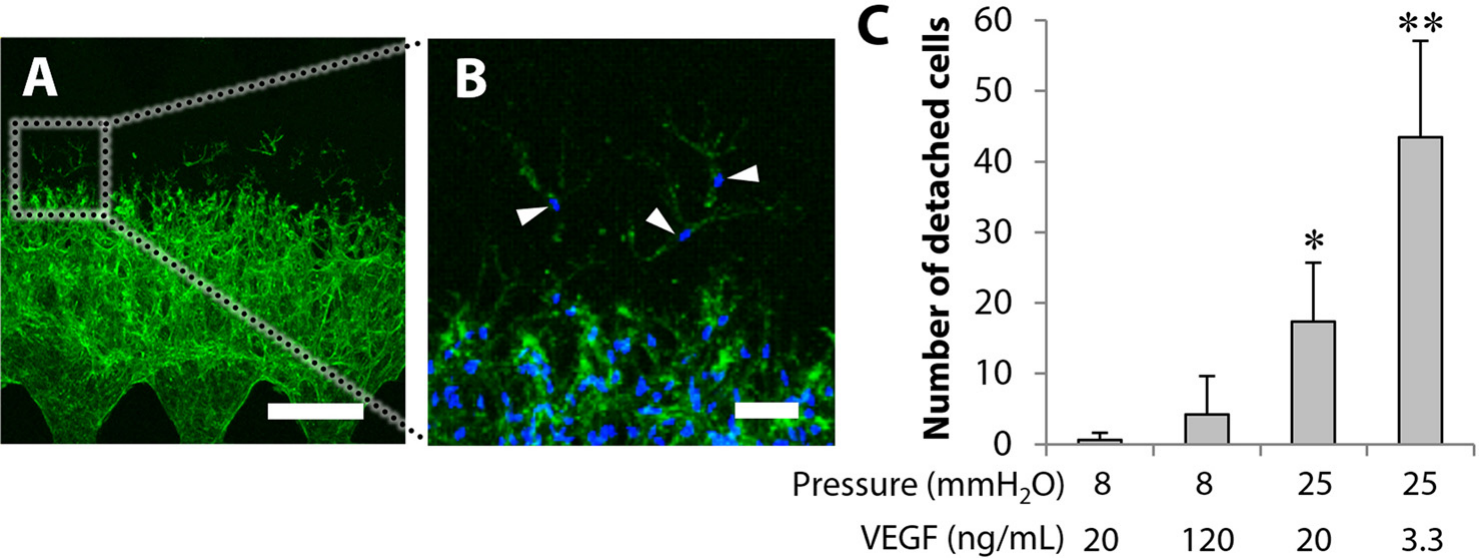
Analysis of cell detachment from network tips. (a) and (b) The representative 3D projected images showing cells (actin, green) and nuclei (blue). Arrowheads indicate the cells detached from network tips. Scale bars, 300 *μ*m (a) and 50 *μ*m (b). (c) Quantitative analysis of the number of detached cells from network tips. Data = mean ± SD (n = 8–10). *P <* *0.05 vs 8 mmH_2_O. **P <* *0.05 vs the other conditions.

Microvascular network formation promoted by interstitial flow was not substituted by the increase in the VEGF concentration. The resulting microvascular networks were different despite the equivalent VEGF flux, and some cells were separated from networks, which was a characteristic phenomenon possibly due to the effect of mechanical stress induced by interstitial flow. Therefore, we analyzed the number of cells detached from network tips [arrowheads, Figs. 5(a) and 5(b)]. The number of detached cells significantly increased in higher interstitial flow conditions (25 mmH_2_O vs 8 mmH_2_O). In particular, the number of detached cells was the greatest in the condition of higher interstitial flow with a lower VEGF concentration such as 25 mmH_2_O conditions with 3.3 ng/ml VEGF [Fig. 5(c)].

### Effect of inhibitors on vascular sprout formation

**FIG. 6.**
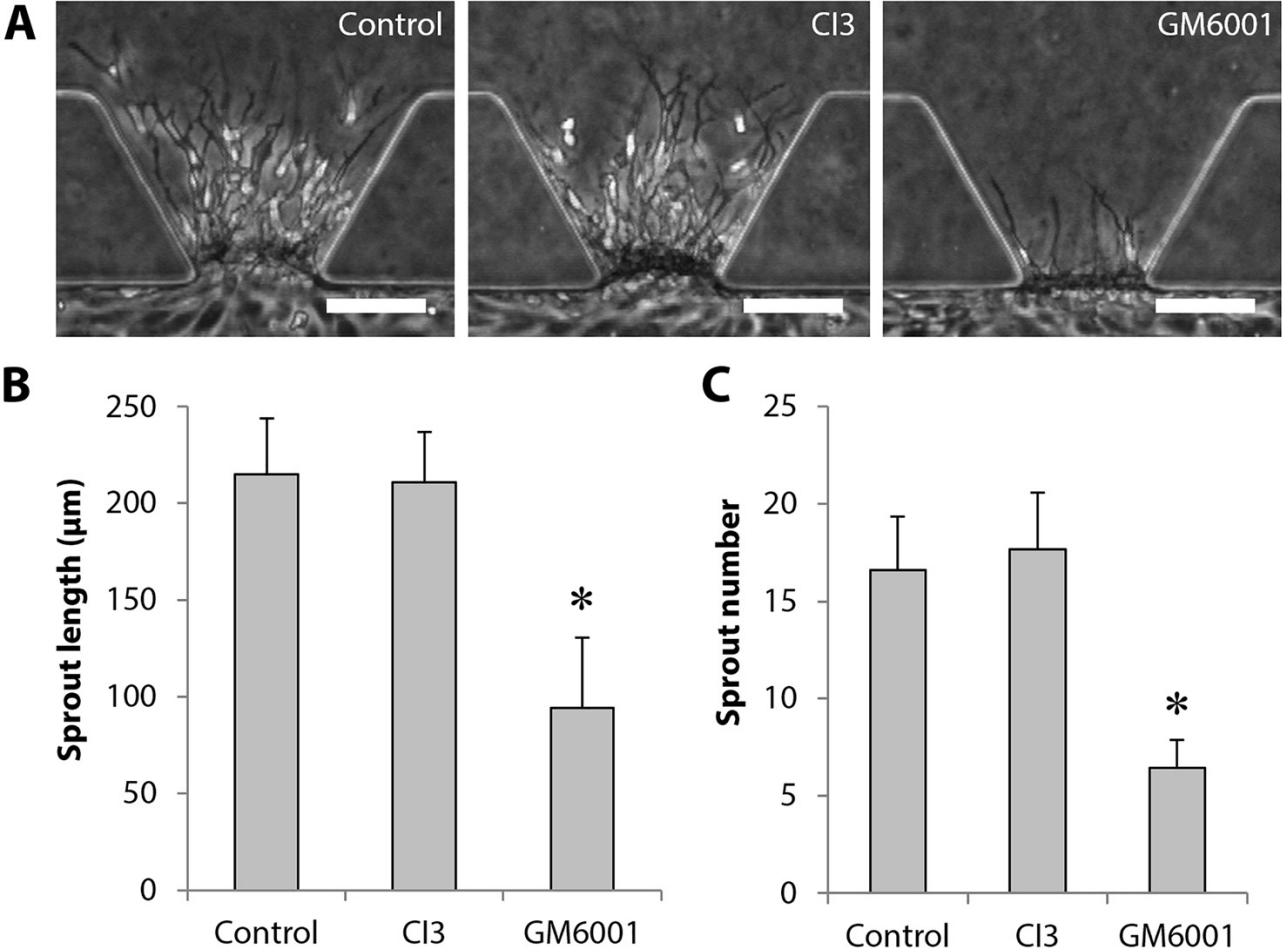
Effect of inhibitors on vascular sprout formation. (a) Representative phase-contrast images of HUVECs on day 1 in control experiments (8 mmH_2_O with 20 ng/ml VEGF), CI3 and GM6001 administration. Scale bars, 100 *μ*m. (b) and (c) Quantitative analysis of the length and number of vascular sprouts, respectively. Data = mean ± SD; n = 12. *P <* *0.05 vs the other conditions.

To explore a mechanism that explains the effect of interstitial flow on angiogenesis, we investigated whether there is a cross talk between interstitial flow- and shear flow-mediated signaling pathways. In particular, we focused on calpain-mediated pathways because calpain is a candidate for regulating the transition of ECs from a quiescent to an invasive phenotype, which is activated by shear stress.^34^ Calpain inhibitors block endothelial alignment in response to shear stress.^35^ In addition, it is reported that an MMP inhibitor, GM6001, and calpain inhibitor III (CI3) significantly attenuated vascular sprout formation of HUVECs induced by shear stress.^18,31^ Therefore, we tested the effects of GM6001 and CI3 on vascular sprout formation in 8 mmH_2_O conditions. The representative phase-contrast images showed that GM6001 significantly attenuated vascular sprout formation, while CI3 failed to attenuate vascular sprout formation [Fig. 6(a)]. These phenomena were also confirmed by quantification of the sprout length [Fig. 6(b)] and sprout number [Fig. 6(c)].

## DISCUSSION

### Microfluidic device that can apply a wide range of interstitial flow magnitudes

In this study, to investigate the mechanism of interstitial flow-induced 3D network formation, we used microfluidic devices that allow the precise control of interstitial flow magnitude. Interstitial flow velocity was controlled by both the liquid level of culture medium between two microchannels and the width of the collagen gel region. Because it is difficult to precisely adjust 2 mmH_2_O manually, we increased the width of the collagen gel region to reduce interstitial flow velocity. Consequently, we could apply interstitial flow velocity ranging from 0.07 to 4.3 *μ*m/s. This interstitial flow magnitude covers a wide range of interstitial flow including physiological ranges from 0 to 2.0 *μ*m/s, with an average of 0.6 ± 0.2 *μ*m/s, which were measured *in vivo.*^36^ In a previous study, Shirure *et al.*^23^ tested 6 and 12 *μ*m/s interstitial flow conditions and these conditions also promoted vascular formation in coculture of ECs and fibroblasts. On the other hand, we focused on a smaller range of interstitial flow and found that both sprout formation and capillary extension were enhanced as we increased the magnitude of interstitial flow ranging from 0.07 to 4.3 *μ*m/s.

Interstitial flow has attracted attention as an important hemodynamic factor controlling angiogenic processes.^12,21,22^ In particular, Vickerman and Kamm^21^ investigated the effect of interstitial flow on angiogenesis focusing on the initial stage and revealed that directional interstitial flow, which was applied from the basal side to the apical side of an endothelial monolayer, promoted vascular sprout formation. In the present experiment, we cultured HUVECs in interstitial flow conditions until day 5 to further investigate the following stage of angiogenesis such as 3D microvascular network formation. Furthermore, we also modified the microfluidic device reported by Vickerman and Kamm,^21^ which allowed us to apply a wide range of interstitial flow magnitudes, and investigated how magnitude of the basal-to-apical interstitial flow contributed to 3D network formation. This study demonstrates that HUVECs formed not only vascular sprouts but also subsequent 3D microvascular networks depending on interstitial flow magnitude.

### Vascular sprout formation depends on interstitial flow magnitude

We demonstrated that HUVECs responded to interstitial flow on day 1 as HUVECs formed more vascular sprouts with increasing interstitial flow magnitude. This is consistent with a recent study which reported that vascular sprout formation of lymphatic cells was enhanced by increasing interstitial flow.^37^ Vickerman and Kamm^21^ also demonstrated that vascular sprout formation was promoted by basal-to-apical interstitial flow, which was related to Src phosphorylation and subsequent VE-cadherin disruption. Interstitial flow application in the present study also induced the Src phosphorylation. We found that Src was activated even in 2 mmH_2_O conditions, the lowest interstitial flow, while no significant difference in the Src expression was detected among 2, 8, and 25 mmH_2_O conditions. The phosphorylation of Src disrupts VE-cadherin which is an intercellular adhesion protein and induces actin polymerization and stress fiber formation.^30,38,39^

The other early stage response of HUVECs to the interstitial flow application was the morphological change. In contrast to the Src expression, HUVECs in a monolayer elongated toward the collagen gel region depending on the flow magnitude. This can be explained by two possible factors. First, HUVECs might be aligned in the flow direction due to the endothelial response to shear stress.^40^ The elongation of the cell structure, which relates to the promotion of directional migration, is induced by mechanical forces such as a high shear stress of ∼0.8 Pa.^41^ This morphological change is accompanied by cytoskeletal reorganization, with actin filaments rearranged and directionally aligned by shear stress.^42^ Second, HUVECs might be aligned in the direction of migration. Because the cells on the surface of a collagen gel migrated into the gel to form vascular sprouts, the cells around vascular sprouts migrated toward the gel region to follow the sprouting cells.

The interstitial flow forces that cells, such as tip cells, would encounter within the interstitium were estimated by Wang and Tarbell.^43^ The shear stress *τ* on the cell surface can be estimated from interstitial flow, which is expressed as follows:

$$\tau \approx \mu \frac{Q}{A}\frac{1}{\sqrt{K}},$$

where *μ* is the fluid viscosity, *Q* is the volumetric flow rate, *A* is the average surface area of collagen gel, and *K* is the Darcy permeability. Based on this equation, the shear stress on the surface of cells within gel, which was derived from a series of interstitial flow velocities generated by 2, 8, and 25 mmH_2_O, can be calculated to be 5.85 × 10^−4^, 5.15 × 10^−3^, and 2.16 × 10^−2 ^Pa, respectively (for details, see the supplementary material). Ueda *et al.*^14^ reported that a shear stress of 0.3 Pa induced the angiogenic response of ECs in a 3D angiogenesis model. In addition, Kang *et al.*^18^ reported that a shear stress of >0.13 Pa significantly induced the angiogenic response of HUVECs. However, the shear stress values derived from interstitial flow in the present study are much smaller than those reported to promote angiogenesis. These results suggest that angiogenic responses to interstitial flow and shear stress can be explained by different mechanisms as discussed below. Our findings suggest that HUVECs can sense interstitial flow in 2, 8, and 25 mmH_2_O conditions and mediate cytoskeletal reorganization through the phosphorylation of Src and EC elongation and that this leads to vascular sprout formation.

### 3D network formation depends on interstitial flow magnitude

Our findings indicate that 3D microvascular network formation is also enhanced by interstitial flow in a magnitude-dependent manner. In particular, HUVECs cultured in 8 mmH_2_O conditions induced the most stable network formation on day 5. Although the highest interstitial flow in 25 mmH_2_O conditions induced the most sprout numbers, the area of degraded gel was also the greatest in this condition, suggesting that the 25 mmH_2_O interstitial flow over-activated HUVECs. Chary and Jain^36^ reported that the interstitial flow velocity from the interstitium into a postcapillary venule ranges in magnitude up to 1.5 *μ*m/s with an average of ∼0.5 *μ*m/s in normal granulation tissue in the rabbit ear chamber. This velocity is close to that generated in 8 mmH_2_O conditions in the present study (0.7 *μ*m/s), suggesting that the interstitial flow magnitude within a physiological range induced stable network formation. Similarly, Kang *et al.*^18^ demonstrated that a shear stress within a physiological range induced angiogenic response. These results suggest that it is important to regulate the magnitude of interstitial flow within a physiological range for enhancing 3D network formation.

The magnitude of interstitial flow affects the balance of convective and diffusive mass transport, which can be estimated by the Peclet number (*Pe*). Hsu *et al.*^44^ reported interesting phenomena that vasculogenesis can be independently stimulated by interstitial flow (*Pe* > 10) or hypoxic conditions (*Pe* < 0.1) and not by the intermediate state (0.1 < *Pe* < 10). In the present study, we also calculated *Pe*. The interstitial flow conditions of 2, 8, and 25 mmH_2_O in the present study correspond to *Pe* values of 18, 92, and 561 (for details, see the supplementary material). Therefore, our experimental conditions were included in the range of *Pe* > 10, where vascular formation was promoted by interstitial flow. This is consistent with our results, while the significant finding in the present study is that the interstitial flow plays an important role in the regulation of both vascular sprout formation and capillary extension in a magnitude-dependent manner.

### Balance of interstitial flow and VEGF modulates 3D network formation

We demonstrated that the proangiogenic effect of interstitial flow application could not be substituted by the increased VEGF concentration. For example, VEGF flux in 8 mmH_2_O conditions with 20 ng/ml VEGF was equivalent to that in 25 mmH_2_O conditions with 3.3 ng/ml VEGF. However, microvascular networks in these conditions exhibited a different morphology. This can be explained that the different morphology was due to the mechanotransduction of interstitial flow, which is independent of VEGF signaling pathways. Both the VEGF concentration and interstitial flow magnitude are important for physiological network formation. Although the VEGF dose-dependent network formation has been demonstrated, the relationship between the VEGF concentration and interstitial flow magnitude was poorly understood.

In a previous study, Shirure *et al.*^23^ reported that microvascular formation in an angiogenesis model was promoted in the upstream of interstitial flow with VEGF stimuli. The enhanced vascular formation in the upstream of interstitial flow is consistent with our findings. However, since they tested only 2 ng/ml VEGF, it remains unclear that how the balance between the VEGF concentration and interstitial flow affects microvascular formation. On the other hand, we tested 3.3, 20, and 120 ng/ml VEGF in combination with interstitial flow, especially focusing on VEGF flux. Our findings indicate that the balance between interstitial flow and the VEGF concentration needs to be considered for constructing desired microvascular networks.

To understand the balance between the interstitial flow magnitude and the VEGF concentration, it might be important to consider effects of these factors on different EC phenotypes: endothelial tip, stalk, and phalanx cells.^45^ Our results suggest that the interstitial flow application promoted the migration of tip cells, which led to network extension, while VEGF promoted the proliferation of stalk cells. Therefore, tip cells were detached from stalk cells in 25 mmH_2_O conditions with 3.3 ng/ml VEGF because tip cell migration was promoted by the highest interstitial flow velocity, while stalk cells could not proliferate due to the low VEGF concentration.

In 8 mmH_2_O conditions, the increase in VEGF from 20 to 120 ng/ml resulted in attenuated vascular formation, which is contrary to the fact that increased VEGF promotes angiogenesis. However, this can be explained if VEGF preferentially affected the enlargement of vasculatures in this condition. Although the network length decreased at 120 ng/ml VEGF conditions, ECs formed locally denser networks compared to 20 ng/ml VEGF conditions. In addition, a recent study by Pontes-Quero *et al.*^46^ reported that high mitogenic stimulation induced by VEGF, or Notch inhibition, induced the cell-cycle arrest of stalk cells and compromised the subsequent proliferation and development of angiogenic vessels.

A synergistic effect of interstitial flow and VEGF supply was notable in terms of MMP activity, which was confirmed by the greatest value in the area of degraded gel in 25 mmH_2_O conditions with 20 ng/ml VEGF. In this condition, MMP activity appeared to be over-activated, which led to the shortest network length. On the other hand, interstitial flow magnitude within the physiological range and moderate VEGF concentration, which is 8 mmH_2_O conditions with 20 ng/ml VEGF, resulted in network formation with the greatest network length. This can be explained if the migration activity of tip cells and proliferation activity of stalk cells are well-balanced in this condition. Our results suggest that the balance between the interstitial flow magnitude and the VEGF concentration plays an important role in the regulation of 3D microvascular network formation *in vitro*.

### Mechanism of the HUVEC response to interstitial flow application

Our experiments using an MMP inhibitor GM6001 and a calpain inhibitor CI3 confirmed that vascular sprout formation was partially dependent on MMP activity but the angiogenic response to interstitial flow was not mediated by calpain. Calpain is a Ca^2+^-dependent intracellular cysteine protease which is activated by shear stress^34,47^ and plays important roles in shear stress-induced mechanotransduction during EC migration.^48^ Specifically, calpain is activated to degrade focal adhesions at the rear of a migrating EC, while lamellipodial protrusion and the formation of new focal adhesions are promoted at the front of the cell, which leads to directional EC migration induced by shear stress.^48,49^ In addition, Kang *et al.*^31^ demonstrated that calpain inhibition significantly inhibited vascular sprout formation induced by shear stress. Therefore, there is increasing evidence that calpain plays important roles in the angiogenic response induced by shear stress. However, since vascular sprout formation induced by interstitial flow in the present study was not mediated by calpain, this suggests that mechanotransduction of interstitial flow may be different from that of shear stress.

It was demonstrated that the forces required to balance drag imparted on the cell by interstitial flow induce a transcellular gradient in cell–ECM adhesion tension, and the tensile stresses at the upstream side of the cell led to the formation of protrusions.^50^ Similarly, interstitial flow produces mechanical stress at cell–ECM interfaces through adhesion molecules such as integrins, which led to focal adhesion kinase-mediated signaling, Src-mediated cell–cell junction remodeling, and finally sprouting angiogenesis.^21^ A similar mechanism might mediate angiogenic responses depending on the magnitude of interstitial flow in the present study.

In conclusions, we investigated microvascular formation using an *in vitro* angiogenesis model focusing on the balance between the interstitial flow magnitude and the VEGF concentration. One of the significant findings in the present study is that the interstitial flow, ranging from 0.07 to 4.3 *μ*m/s, plays an important role in the regulation of both vascular sprout formation and capillary extension in a magnitude-dependent manner. In addition, we demonstrated that neither interstitial flow nor VEGF concentration is sufficient for physiological network formation and that, instead, the balance between them needs to be considered. Our findings are of great interest in terms of vascular tissue engineering and understanding normal and pathological processes such as wound healing and tumor growth.

## METHODS

### Preparation of microfluidic device

**FIG. 7.**
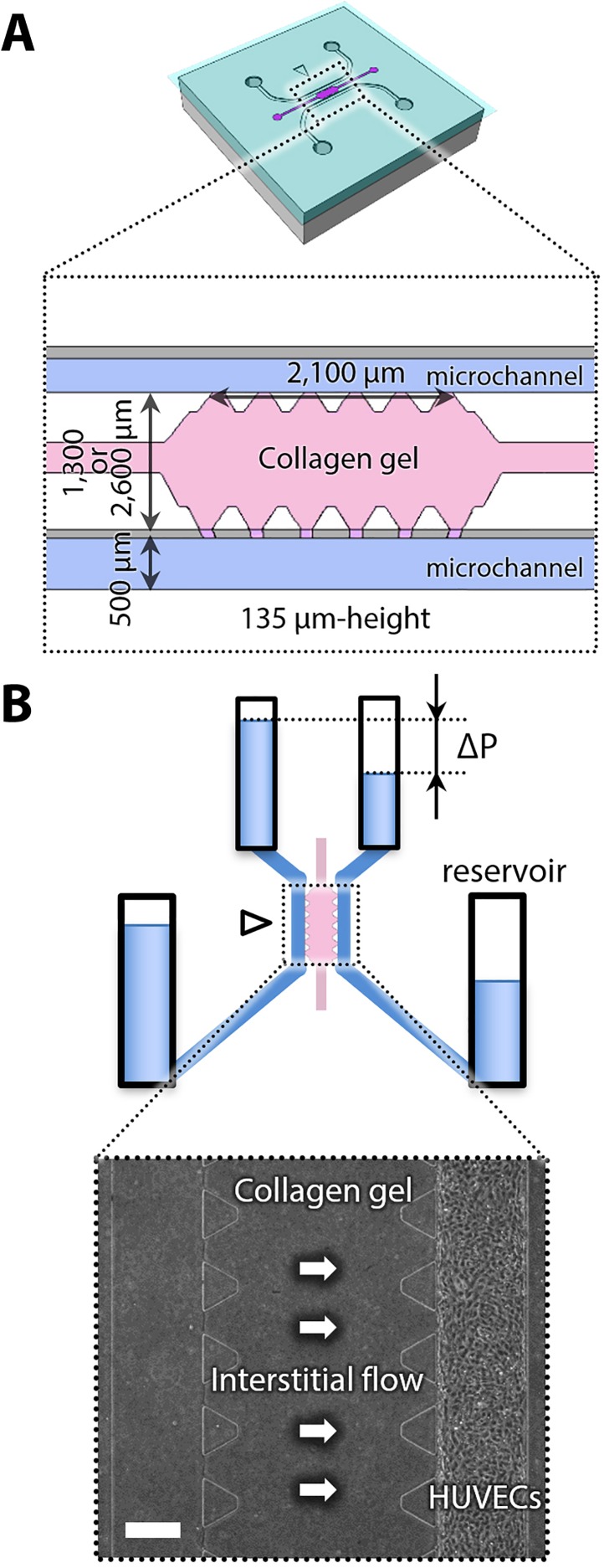
Schematic illustrations of a microfluidic device. (a) Overall and enlarged views of a microfluidic device. (b) An illustration of reservoirs connected to the outlets of microchannels and a phase-contrast image of the gel region. The height difference of culture medium in the reservoirs generates the pressure difference (Δ*P*), resulting in the generation of interstitial flow across the collagen gel. Scale bar, 300 *μ*m.

Microfluidic devices were made of polydimethylsiloxane (PDMS; Dow corning) with cover glass, which have two microchannels separated by a gel region [Fig. 7(a)]. We prepared two types of gel widths, 1300 and 2600 *μ*m, to control interstitial flow magnitude in combination with the pressure difference between the microchannels (Fig. 7).

To create PDMS microfluidic devices, SU-8 photoresist was deposited on a silicon wafer and patterned using photolithography. PDMS was poured onto the SU-8 master mold and cured for >2 h at 85 °C. Cured PDMS was removed from the mold, which was then trimmed and punched. After autoclave sterilization and drying, PDMS devices were bonded to cover glass by using plasma treatment for 90 s to make the adhesion surface between the PDMS device and cover glass hydrophilic. After sterilization by the UV rays for 30 min in the clean bench, PDMS devices were immediately treated with 1.0 mg/ml poly-d-lysine (PDL) solution (Sigma) and placed for >12 h in a humidified 5% CO_2_ incubator at 37 °C. Then, PDL solution was removed and the microfluidic devices were washed with sterilized ion-exchanged water twice and completely dried in an oven for at least 24 h to return PDMS devices to hydrophobic nature. Next, 3.0 mg/ml collagen solution at pH 7.4 was prepared by mixing with collagen (rat tail-type I; BD Biosciences), fibronectin (10 μg/ml; Sigma), 0.1 N NaOH, ion-exchanged water, and 10 × phosphate buffered saline (PBS) on ice and injected into the central channel through collagen filling ports and polymerized for 30 min in the humidified 5% CO_2_ incubator at 37 °C. Before seeding ECs, fibronectin solution (50 *μ*g/ml) was injected into side channels to improve adhesion of ECs, placed for >12 h in the humidified 5% CO_2_ incubator at 37 °C and subsequently replaced with culture medium, EGM-2 BulletKit (Lonza) without VEGF.

### Cell culture

Human umbilical vein endothelial cells (HUVECs; Lonza) were cultured in EGM-2 BulletKit medium containing VEGF and used at passage 6 in our experiments. When the cells reached 80%–90% confluence in dishes, they were trypsinized and seeded onto the upper (PDMS) and lower (cover glass) surfaces of a microchannel, respectively, by injecting 30 *μ*l of cell suspension at 3.0 × 10^6^ cells/ml. EGM-2 BulletKit without VEGF was used to inhibit endothelial sprouting until starting experiments. PDMS solution sterilized by the UV exposure for 30 min was then poured onto the top of collagen filling ports and cured to close their ports. Subsequently, the cells on the channel reached confluence 24–48 h after seeding [Fig. 7(b)]. Ethics approval is not required for this study because we used commercially available cells.

### Interstitial flow experiments

Experiments were started after HUVECs became confluent in a microchannel, which is defined as “Day 0.” Culture medium was then supplemented with 20 ng/ml VEGF (isoform 165, R&D System, Minneapolis, MN) to induce microvascular network formation. Plastic tubes with a 3.0 mm inner diameter, which were used as reservoirs, were connected to the microchannels and the liquid level of culture medium in the reservoirs was adjusted to generate the pressure difference between two microchannels, resulting in the application of interstitial flow across the collagen gel region [Fig. 7(b)]. The pressure differences of 2, 8, and 25 mmH_2_O were used for interstitial flow experiments. Cells were also cultured in static conditions as control experiments. Culture medium was exchanged every day, and the pressure differences were restored (Fig. S1). To further investigate the flow profiles, the gel region was modeled and solved by analysis of the Darcy-Brinkman equation using COMSOL (for details, see the supplementary material). The results demonstrated that the flow was concentrated at the gel surface between PDMS posts, and the average velocity in 2, 8, and 25 mmH_2_O was calculated to be 0.07, 0.61, and 4.02 *μ*m/s, respectively (Fig. S2).

**TABLE I.** Interstitial flow conditions.

Pressure, Δ*P* (mmH_2_O)	Collagen width (*μ*m)	Permeability, *K* (10^−15^ m^2^)	Velocity, *v* (10^−2^ *μ*m/s)
0.0	1300	N/A	0
2.0	2600	6.90	7.1 ± 0.5
8.0	1300	8.88	70.9 ± 6.8
25.0	1300	18.81	431.8 ± 82.7

### Imaging and analysis of 3D network structures

The morphological changes of HUVECs in response to the interstitial flow were monitored and photographed at 24-h intervals for 5 days using a phase-contrast microscope. We then focused on sprout formation after loading interstitial flow for 24 h to evaluate early stages of microvascular formation. The number of vascular sprouts formed between trapezoidal PDMS posts was counted.

Next, morphological changes of ECs were visualized by immunofluorescence staining. In particular, we investigated the expression of Src which functions as a molecular switch of the intracellular signaling pathway during the angiogenic process, including EC proliferation and migration.^20,30^ After loading interstitial flow for 24 h, ECs were fixed with 4% paraformaldehyde for 15 min and rinsed with PBS three times. Cells were then permeabilized in 0.1% Triton X-100 for 20 min and treated with Block Ace (Dainippon Pharmaceutical) for 1 h to inhibit nonspecific staining. Src and platelet endothelial cell adhesion molecule (PECAM) were, respectively, stained with rabbit anti-pSrc polyclonal antibody (Anti-Src [pY^418^], Invitrogen) and mouse antihuman PECAM monoclonal antibody (Millipore) for 2 h. Nuclei were also stained with 4′,6-diamidino-2-phenylindole (DAPI; Invitrogen) for 10 min. Z-stack fluorescence images were obtained using a confocal laser-scanning microscope (LSM700, Carl Zeiss).

Furthermore, the effect of interstitial flow on the morphological changes of ECs in a monolayer was investigated using the confocal images of PECAM. The shape index was calculated to quantify the degree of EC alignment in response to interstitial flow. This value was calculated using the area and perimeter of ECs, and the formula is given as

$$\mathrm{Shape}\;\mathrm{index}=4\pi \;\left(\mathrm{Area}/\mathrm{Perimete}r^{2}\right).$$

A shape index value of 1 indicates a perfect circle. As the value approaches 0, it indicates an increasingly elongated ellipse.

Finally, we analyzed the structures of 3D networks after 5 days of interstitial flow loading. ECs were fixed as described above, and F-actin and nuclei were stained with Alexa Fluor 488-conjugated phalloidin (Invitrogen) for 2 h and DAPI for 10 min, respectively. Fluorescence images were obtained with a confocal microscope and used for quantitative analyses. The length of networks was determined as the length from the cell monolayer to the network tip. The area of degraded gel was measured using two-dimensional (2D) projected images of F-actin. The network density was calculated using 2D projected images of F-actin according to the following formula:

$$\begin{array}{c} D\mathrm{ensity}\;\mathrm{of}\;\mathrm{networks}\left(\%\right)=\left[\left(\mathrm{Area}\;\mathrm{of}\;F-\mathrm{actin}\;\mathrm{in}\;\mathrm{gel}\;\mathrm{region}\right)\;-\;\left(\mathrm{Area}\;\mathrm{of}\;\mathrm{degraded}\;\mathrm{gel}\right)\right]\\ /\left(\mathrm{Area}\;\mathrm{of}\;\mathrm{gel}\;\mathrm{region}\right)\times 100. \end{array}$$

### Calpain and matrix metalloproteinase (MMP) inhibition assay

Calpain is an intracellular calcium-activated cysteine protease, thereby inducing MMP expression needed for angiogenic sprouting.^31^ Calpain inhibition significantly decreased sprout formation induced by wall shear stress and sphingosine 1-phosphate. In the present experiment, we investigated whether calpain also mediated vascular formation induced by interstitial flow. Calpain inhibitor III (CI3; Biolog), which inhibits calpains 1 and 2, and an MMP inhibitor GM6001 (EMD Biosciences) were dissolved in dimethyl sulfoxide (DMSO) at 65.36 and 12.87 mM concentrations, respectively. ECs were preincubated with 50 *μ*M CI3 or 10 *μ*M GM6001 for 1 h before interstitial flow experiments. After loading 8 mmH_2_O interstitial flow for 24 h, vascular sprouts were photographed using a phase-contrast microscope. The length of vascular sprouts was measured using ImageJ. In addition, the number of vascular sprouts formed between trapezoidal PDMS posts was counted. Because 0.08% DMSO was included in culture medium during inhibition experiments, DMSO at the same concentration was also added in culture medium of a control group.

### Statistical analysis

Data are presented as the means ± standard deviation (SD) for each group. Statistical analysis was performed using SPSS software (IBM). At least three independent experiments were performed for all analyses. Comparisons among three or more groups were performed by one-way analysis of variance (ANOVA) followed by posthoc pairwise comparison testing using Tukey's method. Two-way ANOVA was only performed to determine the effect of effective VEGF supply on microvascular network formation. A value of *p *<* *0.05 was considered to indicate statistical significance.

## SUPPLEMENTARY MATERIAL

See the supplementary material for the details of interstitial flow analysis.
